# Rotation of skylight polarization during learning walks is necessary to trigger neuronal plasticity in *Cataglyphis* ants

**DOI:** 10.1098/rspb.2021.2499

**Published:** 2022-01-26

**Authors:** Robin Grob, Oliver Holland Cunz, Kornelia Grübel, Keram Pfeiffer, Wolfgang Rössler, Pauline N. Fleischmann

**Affiliations:** Behavioural Physiology and Sociobiology (Zoology II), Biocentre, University of Würzburg, 97074 Würzburg, Germany

**Keywords:** celestial compass calibration, central complex, memory formation, mushroom bodies, solar ephemeris

## Abstract

Many animals use celestial cues for impressive navigational performances in challenging habitats. Since the position of the sun and associated skylight cues change throughout the day and season, it is crucial to correct for these changes. *Cataglyphis* desert ants possess a time-compensated skylight compass allowing them to navigate back to their nest using the shortest way possible. The ants have to learn the sun's daily course (solar ephemeris) during initial learning walks (LW) before foraging. This learning phase is associated with substantial structural changes in visual neuronal circuits of the ant's brain. Here, we test whether the rotation of skylight polarization during LWs is the necessary cue to induce learning-dependent rewiring in synaptic circuits in high-order integration centres of the ant brain. Our results show that structural neuronal changes in the central complex and mushroom bodies are triggered only when LWs were performed under a rotating skylight polarization pattern. By contrast, when naive ants did not perform LWs, but were exposed to skylight cues, plasticity was restricted to light spectrum-dependent changes in synaptic complexes of the lateral complex. The results identify sky-compass cues triggering learning-dependent versus -independent neuronal plasticity during the behavioural transition from interior workers to outdoor foragers.

## 1. Background

**Figure 1.**
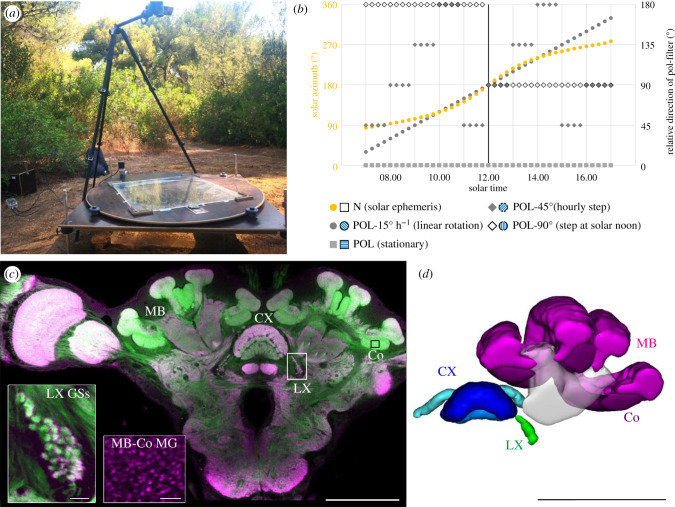
Experimental procedure. (*a*) A rotating table was installed 30 cm above the natural nest entrance of *Cataglyphis nodus*. In the opening in the middle of the table, a linear polarization filter was placed. Novices that were allowed to perform LW did so under the restricted area (60 × 60 cm) that was covered by the filter. (*b*) The linear polarization filter (POL) was rotated over the day in relation to the natural rotation of the sky polarization pattern (N, yellow circles (for the Schinias National Park on the 1st of August 2019)). The filter was either turned once a day at solar noon (POL-90°, white diamond), hourly (POL-45°, grey diamond) or continuously (POL-15° h^−1^, grey circles), or was stationary over the day (POL, grey square). (*c*) Brains of ants that participated in the field experiments were double labelled with anti-synapsin antibodies (magenta) and f-actin labelling with fluor-phalloidin (green) (Scale bar, 200 µm). In this study, we focused on neuropils along with two major visual pathways: the central complex (CX), the mushroom body (MB) collar (Co) microglomeruli (MG) (MB-Co MG; black box and inset) and giant synapses in the lateral complex (LX GS; white box and inset). Scale bar insets = 10 µm. (*d*) Three-dimensional reconstruction of MB (magenta) with the Co in dark magenta cut open to highlight the lateral-complex structures (green) located posterior to the peduncle. CX in shades of blue (Scale bar, 200 µm). (Online version in colour.)

Finding the way back home is a crucial challenge that many animals face. Desert ants of the genus *Cataglyphis* use an impressive set of navigational cues to get back to their nest even under exceptionally harsh conditions. To spend as little time as possible in the blazing heat of the sun, *Cataglyphis* foragers steer back to their nest in the shortest way possible with astonishing accuracy [1]. The ants integrate directional information from a celestial compass and distance information from a step integrator into a vector pointing homewards [1]. However, a compass based on celestial cues comes with its own challenges. Both the position of the sun and the associated sky polarization pattern change throughout the day. The ant's internal skylight compass must compensate for this movement, especially during extended foraging trips, or upon subsequent visits to a profitable food site. This is especially important around solar noon when the sun's horizontal position (azimuth) changes most rapidly (figure 1). The sun's daily course (solar ephemeris) depends on the season and geographical position, making the problem even more complex. Due to these unpredictable variables, the inherent knowledge about the solar ephemeris is very limited [2]. Hence, a celestial compass must be learned before embarking upon far-ranging foraging journeys [2–5]. Desert ants perform well-structured initial learning walks (LW) during the transition phase from interior worker to outside forager [6,7]. *Cataglyphis nodus* repeatedly stop their forward movement to perform characteristic turns [6] and stops with views directed towards their nest entrance. During this behaviour, naive ants do not rely on the celestial compass [4], but rather use the earth's magnetic field as directional input for path integration [8]. Full body turns have been suggested to be used to read out the celestial compass [6], for example by taking skylight snapshots [9]. Here, we investigate how the rotation of the sky influences neuroplasticity during the initial LW of *C. nodus*. Previous studies have shown that initial LW trigger structural synaptic plasticity in two visual pathways to integration centres in the ant brain [3,4,10]. The central complex (CX) [11] and mushroom bodies [12,13] are essential neuropils involved in memory-based orientation and navigation [14]. The CX has been suggested to be the neuronal correlate for path integration [15], whereas the mushroom bodies circuits have the capacity for storing view-based visual information [16].

In the present study, we ask whether the early experience of changing sky polarization patterns represents the necessary cue to induce volumetric and structural synaptic changes (neuronal plasticity) in integration centres along two major visual pathways. The results demonstrate that the experience of a rotating sky polarization pattern during the performance of initial LW in the natural habitat is necessary to trigger calibrations in neuronal circuits of the CX and mushroom bodies. This suggests that the natural rotation of the sky polarization pattern has to be acquired during this early learning phase.

## 2. Methods

### (a) Experimental model and subject details

Four ant colonies of *Cataglyphis nodus* (Brullé 1832) in the pine forest of Kotychi-Strofylia National Park, Lapas, Greece and Schinias National Park, Marathonas, Greece were used in the summer of 2019 for this study. All ants outside the nest were marked with car paint for 3 consecutive days before the experiments started (Motip Lackstift Acryl, MOTIP DUPLI GmbH, Haßmersheim, Germany). This made sure that only unmarked novices that had not performed LW before the experiments were included in the experiments [17].

### (b) Experimental procedure

#### (i) Light exposure

Interior workers were collected from two colonies that were excavated at night under a tent using red light in the Kotychi-Strofylia National Park (Strofylia-1, Strofylia-2); for more details see electronic supplementary material). All foragers of the colonies had been marked over 3 consecutive days beforehand. The ants were kept in a dark box until the next day. Interior workers (unmarked ants) were apportioned to four experimental groups in different boxes. Each group was exposed to light pulses under the natural sky five times a day (07.30, 09.30, 11.30, 13.30, 15.30 solar time) for 45 min each for 3 consecutive days (method established by Schmitt *et al*. [18]). The groups were exposed to different light conditions: full wavelength spectrum of natural sunlight under a UV-light permeable Plexiglas (Plexiglas (GS) 2458, Evonik Performance Materials GmbH, Essen, Germany) (LE-N), full wavelength spectrum of natural skylight with a stationary linear polarization pattern under a UV-light permeable linear polarizer (POL, OUV6060-C-HNP'B, Knight Optical Ltd, Harrietsham, UK) (LE-POL), natural skylight without light of wavelengths shorter than below 420 nm using a UV cut-off filter (Plexiglas (Gallery) 0A570 GT, Evonik Performance Materials GmbH, Essen, Germany) (LE-UVB) and no light within the dark box as a control (DD). All four boxes were otherwise treated identically. After the third day of exposure, the ants were kept in the dark overnight and were subsequently dissected, and the neuroanatomical staining process was started immediately in Greece (see below). This allowed for all groups to be stained at the same time after their experimental treatment.

#### (ii) Learning walk manipulations

**Figure 2.**
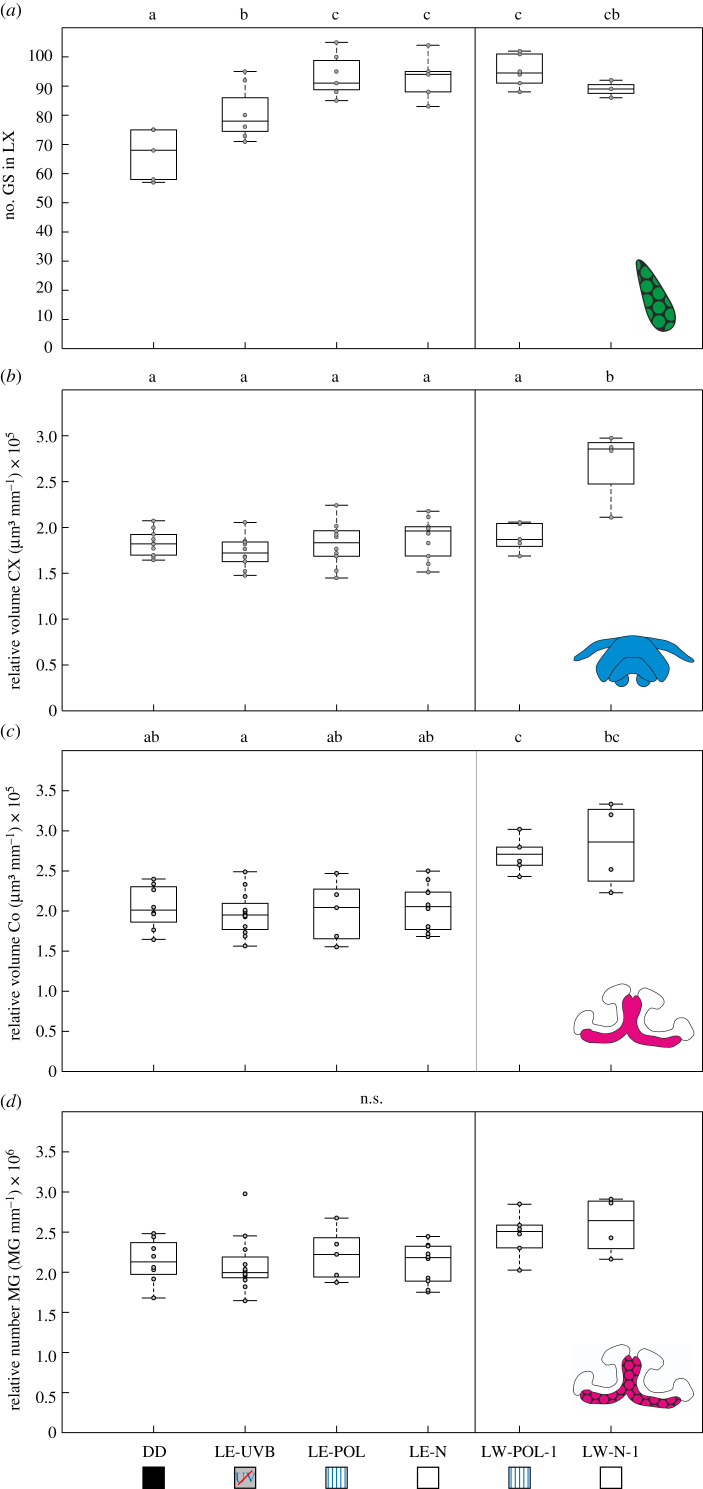
The influence of LE and LW on neuronal plasticity. Neuronal changes after 3 days in complete darkness (DD) compared to LE under skylight with a UV cut-off filter (LE-UVB), under full-spectrum skylight with a linear polarization filter (LE-POL) and under natural skylight (LE-N) as well as after 3 days of LW under full-spectrum skylight with a linear polarization filter (LW-POL-1) and under natural skylight (LW-N-1). (*a*) The absolute number of synaptic complexes is shown for the giant synapses (GS; n_DD_ = 6, n_LE-UVB_ = 8, n_LE-POL_ = 7, n_LE-N_ = 6, n_LW-POL-1_ = 6, n_LW-N-1_ = 4) in the lateral complex (LX). The size-corrected volumes are shown for (*b*) the central complex (CX; n_DD_ = 10, n_LE-UVB_ = 10, n_LE-POL_ = 10, n_LE-N_ = 10, n_LW-POL-1_ = 5, n_LW-N-1_ = 4) and (*c*) the mushroom body collar (Co; n_DD_ = 8, n_LE-UVB_ = 12, n_LE-POL_ = 5, n_LE-N_ = 10, n_LW-POL-1_ = 6, n_LW-N-1_ = 4). (*d*) The size-corrected total number of MG is shown for the mushroom body collar (n_DD_ = 8, n_LE-UVB_ = 12, n_LE-POL_ = 5, n_LE-N_ = 10, n_LW-POL-1_ = 6, n_LW-N-1_ = 4). The central line of each boxplot depicts the median of the data. The upper and lower limits of the boxes show the 25th and 75th percentiles, while the whiskers extend to the extreme data points without outliers. Outliers are values that are more than 1.5 times the interquartile range from the edge of the box. All data points are plotted as grey circles. Significant differences between the groups are marked with lower case letters (Kruskal–Wallis test (*α* = 0.05) and post hoc Mann–Whitney U-test with Benjamini–Hochberg correction (false discovery rate of *α* = 0.05)). For statistical details, see text. (Online version in colour.)

**Figure 3.**
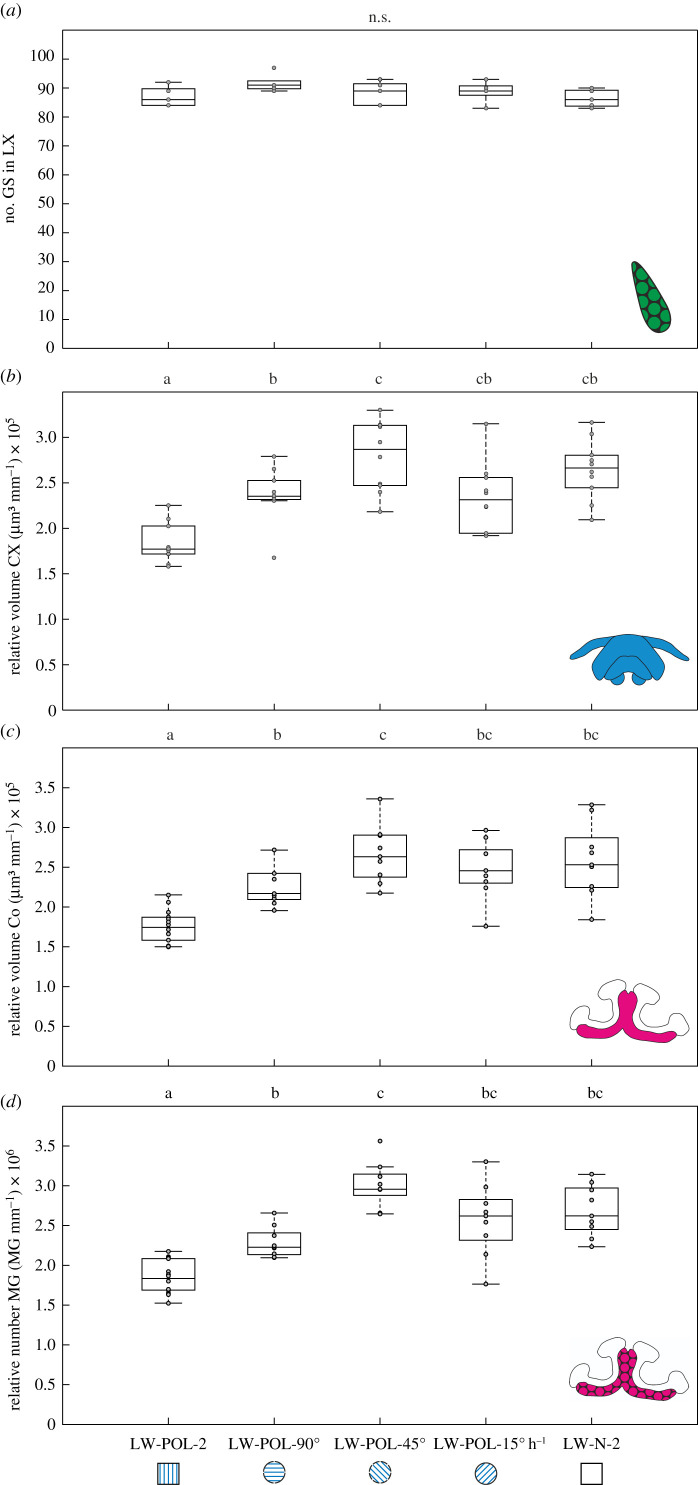
The influence of a rotating polarization pattern on neuronal plasticity following LW. Neuronal changes after 3 days LW under different skylight conditions. The labels indicate LW together with the light filter conditions (either under a polarizer (POL) or under natural light conditions (N)): stationary linear polarization filter (LW-POL-2), linear polarization filter that was turned by 90° once per day at solar noon (LW-POL-90°), linear polarization filter that was turned hourly by 45° (LW-POL-45°), linear polarization filter that was turned constantly at a speed of 15° h^−1^ (LW-POL-15° h^−1^) and the natural skylight (LW-N-2). (*a*) The absolute numbers of the synaptic complexes in the lateral complex (giant synapses, GS; n_LW-POL-2_ = 5, n_LW-POL-90°_ = 5, n_LW-POL-45°_ = 5, n_LW-POL-15° h^−1^_ = 5, n_LW-N-2_ = 5) are shown. The size-corrected volumes are shown for (*b*) the central complex (CX; n_LW-POL-2_ = 10, n_LW-POL-90°_ = 10, n_LW-POL-45°_ = 10, n_LW-POL-15° h^−1^_ = 10, n_LW-N-2_ = 10) and (*c*) the mushroom body collar (Co; n_LW-POL-2_ = 14, n_LW-POL-90°_ =9, n_LW-POL-45°_ = 9, n_LW-POL-15° h^−1^_ = 9, n_LW-N-2_ = 9). (*d*) The size-corrected total numbers of MG are shown for the collar (n_LW-POL-2_ = 14, n_LW-POL-90°_ = 9, n_LW-POL-45°_ = 9, n_LW-POL-15° h^−1^_ = 9, n_LW-N-2_ = 9). For figure conventions, figure 2, and for statistical details, see text. (Online version in colour.)

Naive ants (novices) from two colonies (Strofylia-3 (figure 2), Schinias-1 (figure 3); for more details see electronic supplementary material) were allowed to perform LW in a restricted area around their natural nest entrance (60 × 60 cm restricted by a transparent plastic fence). Only experienced foragers (marked ants) that did not participate in the experiments were allowed to leave the fenced area. The spectrum of skylight the ants were able to perceive during their LW was manipulated with a linear, UV-light permeable polarization filter (60 × 60 cm, figure 1*a*). It was installed in the middle of a custom-made rotating table 30 cm above the nest entrance from the third day of marking. This made sure that ants that did not yet leave the nest but spent time inside the nest entrance could only perceive the experimental condition. To test whether changes in the polarization pattern over the course of the day for a sequence of 3 days were necessary to trigger neuronal plasticity, ants were presented with one of four POL rotations: (1) stationary POL, (2) 90° rotation of POL at solar noon, (3) rotation of POL by 45° every 60 min or (4) a continuously rotating POL at a speed of 15° h^−1^ (figure 1*b*). For the continuous rotation, the rotating table was moved by a stepper motor (Nema 17 stepper motor, stepperonline Inc., New York City, NY, USA) with a micro-stepping controller (SMC11, Nanotec Electronic GmbH & Co. KG, Feldkirchen, Germany). The stepper motor was controlled by an Arduino microcontroller (Genuino ZERO, arduino.cc) using a custom-written script and powered by a customized battery power supply made by the Biocentre's electronic workshop [8]. As a control, ants were allowed to perform LW under natural skylight, including the naturally moving polarization pattern. For both experiments (light exposure (LE, figure 2) and LW under a rotation polarizer (figure 3)), LW control groups under stationary POL (LW-POL-1 (in Strofylia), LW-POL-2 (in Schinias)) and under natural skylight (LW-N-1 (in Strofylia), LW-N-2 (in Schinias)) were collected. Novices were allowed to perform LW under one of these conditions for 3 consecutive days. During the LW, the ants were able to see the position of the sun. On the third day, ants that reached the fence, indicating that they had performed multiple LW previously [10], were caught and kept in a dark box until dissection the next day (method established by Grob *et al*. [4]). Subsequently, all ants outside of the nest were marked again for 3 consecutive days.

### (c) Neuroanatomical procedures

To analyse volumetric and synaptic changes following LE and LW, the ants' brains were double labelled in the field laboratory in Greece using a primary antibody against synapsin (SYNORF1, kindly provided by E. Buchner, University of Würzburg, Germany) and CF633 Phalloidin (00046, Biotium Inc., Fremont, CA, USA) following established protocols from Habenstein *et al*. [19].

The ants were anaesthetized on ice, decapitated, and the brains were immediately dissected under cooled Ringer's solution (127 mM NaCl, 7 mM KCl, 1.5 mM CaCl_2_, 0.8 mM Na_2_HPO_4_, 0.4 mM KH_2_PO_4_, 4.8 mM TES and 3.2 mM trehalose, pH 7.0). The brains were then transferred to a 24-well plate and fixed in 4% formaldehyde in phosphate-buffered saline (PBS) at 4°C overnight. The brains were rinsed in PBS three times for 10 min each. To make the cell membranes permeable for antibody application, the brains were rinsed once with 2% Triton-X 100 for 10 min and twice with 0.5% Triton-X 100 for 10 min. To block unspecific binding sites, the brains were incubated for 1 h at room temperature on a shaker in a 0.5% Triton-X 100 solution in PBS with 2% of normal goat serum (NGS, Jackson Immuno Research Laboratories). Subsequently, they were incubated on a shaker in primary antibody to synapsin from mouse 1 : 50 in PBS with 0.5% Triton-X 100 and 2% NGS for 5 days in a fridge at 4°C. The brains were then rinsed three times in PBS for 20 min and subsequently incubated in a secondary anti-mouse antibody from goat coupled to AlexaFluor 568 (A12380, Molecular Probes, Eugene, OR, USA) dye (1 : 250) and CF633 Phalloidin (2.5 μl Phalloidin from Methanol stock solution in 500 µl PBS) in PBS with 0.5% Triton-X 100 and 1% NGS for 3 days on a shaker at 4°C. After incubation, the brains were rinsed in PBS four times for 20 min each and post-fixed in 4% formaldehyde in PBS overnight at 4°C. They were washed four times in PBS for 20 min each and then dehydrated in an ascending ethanol series (30%, 50%, 70%, 90%, 95% for 3–4 min each, and two times in 100% ethanol for 5 min). The brains were cleared in methyl salicylate (4529.1, Carl Roth Gmbh & Co. KG, Karlsruhe, Germany) and finally transported from the field laboratory to the University of Würzburg for confocal laser scanning microscopy and further analyses.

### (d) Quantification and statistical analysis

#### (i) Data analysis

A confocal laser scanning microscope (Leica TCS SP8, Leica Microsystems GmbH, Wetzlar, Germany) was used for scanning the brains as image stacks at a step size of 5 µm. APO 20×/0.7 IMM water immersion objective with a step size of 5 µm for overviews with 0.75 digital zoom for whole-brain scans, with 1.6 zoom for close-ups of the CX, with 2.0 zoom for the mushroom body calyx and with 4.0 zoom for detailed scans of the lateral complex. A PL APO 63×/1.2 W objective with 2.0 digital zoom with a step size of 0.5 µm was used for high-resolution scans in the collar of the mushroom body calyx.

The neuropils and their subunits can easily be distinguished in anti-synapsin labelled whole mount brains (figure 1*c*). The volumes of the CX, the mushroom body and their subunits were analysed using the three-dimensional reconstruction software TrakEM2 [20] plugin for ImageJ 1.52n (Wayne Rasband, National Institutes of Health, USA).

For analysing structural synaptic plasticity during LE and LW, microglomeruli (MG) were quantified with Amira (Amira-Avizo Software 2019.1, Thermo Fisher Scientific Inc., Waltham, Massachusetts, USA) in the visual subregions of the mushroom body calyx (collar) using the protocol established by Groh *et al*. [21]. Anti-synapsin, phalloidin labelled synaptic boutons were counted in three defined volumes (1000 µm^3^ each) by a person who was blind to the experimental treatments. MG density was calculated by averaging the analysed volumes of the collar as numbers of MG per 1000 µm^3^. The total numbers of MG per collar were estimated by extrapolation of the average MG numbers per 1000 µm^3^ to the total volume of the collar. This represents a good approximation, as MG densities are largely homogeneous throughout the collar of *C. nodus* neuropils [4,22]. Giant synapses in the lateral bulb of the lateral complex in one brain hemisphere of each experimental ant were traced and quantified using TrakEM2 [20] plugin for ImageJ 1.52n (Wayne Rasband, National Institutes of Health, USA) using the protocol established by Grob *et al*. [23].

#### (ii) Statistics

Since *C. nodus* ants’ sizes and, therefore, brain volumes showed differences between the experimental groups, the size of the neuropils was corrected for the ants' sizes (which correlates with the ants’ total brain size), by dividing the volume by the thorax length, which is an established measure for body size [24]. The neuroanatomical differences between the groups were compared using a Kruskal–Wallis test (*α* = 0.05) and a post hoc Mann–Whitney U-test with Benjamini–Hochberg correction [25] (false discovery rate *α* = 0.05). All descriptive statistical analyses were done with MATLAB (2015a or 2018a, The MathWorks Inc., Natick, MA, USA).

### (e) Nomenclature

For the nomenclature of neuropils in the ant brain, we refer to Habenstein *et al*. [19] (see also https://www.insectbraindb.org for 3D data of the *Cataglyphis* brain) and Ito *et al*. [26]. For the terminology related to spatial orientation and navigation, we refer to Grob *et al*. [14].

## 3. Results and discussion

### (a) Light exposure

Celestial compass information provided by the polarization pattern of the skylight, the most important navigation cue for *Cataglyphis* desert ants [1,27], is transferred from the dorsal rim area of the compound eyes and optic lobes to the CX via the anterior optic tract and the bulbs of the lateral complex [3,4,18]. In the lateral-complex bulbs, the last synaptic relay station of the sky-compass pathway before the CX, sensory exposure to manipulated skylight (LE) led to an increase in the number of large synaptic complexes (giant synapses) dependent on the light spectra perceived during first LE (figure 2*a*; Kruskal–Wallis test: number giant synapses: *χ* = 21.29, *n* = 37, *p* = 0.0007; n_DD_ = 6, n_LE-UVB_ = 8, n_LE-POL_ = 7, n_LE-N_ = 6, n_LW-POL-1_ = 6, n_LW-N-1_ = 4). The increase was more prominent when the ants were exposed to the full light spectrum compared to skylight without UV-light or constant darkness (figure 2*a*; numbers giant synapses: median (minimum/maximum): DD = 68 (57/75), LE-UVB = 78 (71/95), LE-POL = 91 (85/105), LE-N = 94 (83/104), LW-POL-1 = 94,5 (88/102), LW-N-1 = 89 (86/92)). The light spectrum-dependent increase in bulb giant synapse numbers is comparable with the increase previously found under similar conditions in *Cataglyphis fortis* [18]. UV-light is an important navigation cue for desert ants, since they only perceive the polarization pattern of the sky in the UV-light spectrum [28]. Interestingly, the increase in the numbers of giant synapses occurred independently of a changing polarization pattern (figures 2*a* and 3*a*) and in the absence of LW behaviour (figure 2*a*). This was different in the CX, the target integration centre of the anterior optic tract downstream from the lateral-complex bulb synapses. Here, a volume increase was triggered only when the ants had been able to experience natural skylight with a changing polarization pattern during 3 days of performing initial LW (figure 2*b*, Kruskal–Wallis test: relative CX volume: *χ* = 14.09, *n* = 49, *p* = 0.0151; n_DD_ = 10, n_LE-UVB_ = 10, n_LE-POL_ = 10, n_LE-N_ = 10, n_LW-POL-1_ = 5, n_LW-N-1_ = 4). Contrary to the lateral-complex bulb synapses, exposure to natural skylight alone was not sufficient to trigger volumetric changes in the CX (figure 2*b*). We hypothesize that structural plasticity in lateral-complex bulb synapses promotes stabilization of the sky-compass network in downstream CX circuits via inhibitory tangential neurons (ring neurons in *Drosophila*) to adapt the neuronal network to new sensory input (homeostatic plasticity) [3,18,29].

Visual information, e.g. about the panorama and the sky, is transferred to the mushroom bodies, mainly via the anterior superior optic tract [3,4,19]. Like in the CX, the volume of the visual input region of the mushroom body calyx (collar) did not increase when the ants were exposed to skylight in the absence of LW. Only when the ants were able to perform LW, the volumes of the mushroom body collar regions increased (figure 2*c*; Kruskal–Wallis test: relative mushroom body volume: *χ* = 20.11, *n* = 45, *p* = 0.0012; n_DD_ = 8, n_LE-UVB_ = 12, n_LE-POL_ = 5, n_LE-N_ = 10, n_LW-POL-1_ = 6, n_LW-N-1_ = 4). The dependency on the early learning behaviour of desert ants suggests that volume-related structural neuronal plasticity in the collar is triggered by learning and long-term memory formation. However, we did not find changes in the relative numbers of synaptic complexes (MG) in the mushroom body calyx (figure 2*d*; Kruskal–Wallis test: 2D: relative number MG: *χ* = 10.48, *n* = 45, *p* = 0.0626; n_DD_ = 8, n_LE-UVB_ = 12, n_LE-POL_ = 5, n_LE-N_ = 10, n_LW-POL-1_ = 6, n_LW-N-1_ = 4). This contrasts with previous results showing that both the numbers of MG and the volumes of the mushroom body collar increase after the performance of LW under natural skylight [4], which is also confirmed by our current experiments using a rotating polarization filter (see below and figure 3). We assume that this difference in mushroom body plasticity might be due to either colony-level variance or other biological variables. The mushroom bodies are essential for processing visual information about the landmark panorama [3,12,13]. Consequently, the quality of panoramic information may exert additional influences on LW-related plasticity in the mushroom bodies. While the LW experiments that revealed a significant influence of the skylight on mushroom body plasticity ([4], and figure 3) were performed in clearings of Schinias National Park, the experiment shown in figure 2*c,d* was performed in the more cluttered environment of Strofylia National Park. This provides an important hint for future experiments to investigate the influence of the panorama on mushroom body plasticity during LW in a more controlled situation. Interestingly, long-term LE (5 days) leads to pruning in MG numbers in *C. fortis* [10], while 3 days of LE in *C. nodus* (figure 2*c*,*d*) did not lead to a significant change in MG numbers. This indicates that LE-related synaptic pruning in the mushroom bodies obviously requires longer stimulus exposure compared to the exposure time required for LW-related increases of mushroom body synaptic complexes [4,6,30].

Overall, this means that homeostatic synaptic plasticity following longer LE occurs along with both visual pathways [3,29]. The majority of tangential neurons projecting from the lateral complex to the CX are GABAergic [18], whereas projection neurons providing input to the mushroom bodies are presumably cholinergic [31]. As a consequence, the increase in giant synapses in the lateral-complex bulb, in contrast with pruning of MG in the mushroom body, could be attributed to different functional properties of the input neurons [3]. Ants are exposed to skylight before they start to perform LW, e.g. while looking out of the nest entrance, or while leaving the nest for seconds during waste disposal or digging [6]. Here, visual pathways could already undergo homeostatic plasticity preparing the ant's brain for the drastic changes in their visual environment.

### (b) Rotation of polarization pattern

Learning-dependent neuronal plasticity in the CX, and in previous experiments also in the mushroom bodies (figure 2 and Grob *et al*. [4]), occurred only when the ants were able to perform LW under natural skylight. It was absent when the polarization pattern was stationary. Therefore, using a linear polarizer (POL), we tested whether rotation of a polarization pattern over the day is a necessary change in skylight cues to trigger neuronal plasticity after LW in a natural habitat (figure 3). As expected from the result shown in figure 2*a*, rotating POL had no influence on the number of lateral-complex bulb giant synapses (figure 3*a*, Kruskal–Wallis test: number giant synapses: *χ* = 5.45, *n* = 25, *p* = 0.2438; n_LW-POL-2_ = 5, n_LW-POL-90°_ = 5, n_LW-POL-45°_ = 5, n_LW-POL-15° h^−1^_ = 5, n_LW-N-2_ = 5; number giant synapses: median(minimum/maximum): LW-POL-2 = 86 (84/92), LW-POL-90° = 91 (89/97), LW-POL-45° = 89 (84/93), LW-POL-15° h^−1^ = 89 (83/93), LW-N-2 = 86 (83/90)). This reaffirms that structural synaptic plasticity in the lateral-complex bulb is independent from both a changing polarization pattern and LW behaviour. By contrast, a single 90° rotation of POL at solar noon, as well as rotating the POL either by 45° every hour, or continuously at a speed of 15° h^−1^ (figure 1*b*) led to a comparable increase in CX volume as under natural skylight (figure 3*b*; Kruskal–Wallis test: relative CX volume: *χ* = 25.23, *n* = 50, *p* < 0.0001; n_LW-POL-2_ = 10, n_LW-POL-90°_ = 10, n_LW-POL-45°_ = 10, n_LW-POL-15° h^−1^_ = 10, n_LW-N-2_ = 10).

Similar to the pattern of results observed in the CX, a single rotation of POL per day triggered a volume increase in the mushroom body collar (figure 3*c*; Kruskal–Wallis test: relative mushroom body volume: *χ* = 10.25, *n* = 50, *p* = 0.0363; n_LW-POL-2_ = 14, n_LW-POL-90°_ = 9, n_LW-POL-45°_ = 9, n_LW-POL-15° h^−1^_ = 9, n_LW-N-2_ = 9). Likewise, the number of MG in the collar increased depending on a rotating POL (figure 3*d*; Kruskal–Wallis test: relative number MG: *χ* = 34.27, *n* = 50, *p* < 0.0001; n_LW-POL-2_ = 14, n_LW-POL-90°_ = 9, n_LW-POL-45°_ = 9, n_LW-POL-15° h^−1^_ = 9, n_LW-N-2_ = 9). The mushroom body plasticity after LW under a changing polarization pattern is in line with previous experiments [4]. The results demonstrate that the moving skylight polarization pattern during initial LW represents the necessary skylight cue for triggering learning-dependent changes in visual integration centres in the *Cataglyphis* brain associated with sky-compass processing. By contrast, the movement of the sun without a (moving) polarization pattern is not sufficient to trigger the LW-related neuronal changes in both visual integration centres. This hierarchy in cue importance is reflected in the orientation behaviour of experienced foragers. *Cataglyphis* foragers preferentially rely on directional information provided by the polarization pattern of the sky compared to the position of the sun [27]. While the direct position of the sun and the polarization pattern are detected in different parts of the eye [3], directional information from the two sources of information are processed by the same neurons in the anterior optic tubercle of the insect brain [32–35]. In the CX, directional information from the sun and the polarization pattern of the sky are integrated to complement each other to provide a robust celestial compass [15,33].

### (c) Acquisition of the solar ephemeris

Our results support the hypothesis that *Cataglyphis* ants use the changing sky polarization pattern to learn the solar ephemeris during their initial LW outside the nest [3,4,6,7,36]. Desert ant foragers rely on a time-compensated sun compass during their long and far-ranging foraging runs, for which they have to learn and memorize the seasonal course of the sun over the day [2,5,36]. This is also crucial for repeated visits to the same food source over extended periods. The transition from dark-adapted interior worker to visually guided forager in bright skylight requires the calibration of the ants' visual guidance systems [3,29]. During their LW, *C. nodus* ants perform a specific turn besides pirouettes, so-called voltes, that may allow them to read out the sky polarization pattern [6]. This is supported by the fact that closely related *C. fortis,* living in almost featureless environments, exclusively perform voltes, but not pirouettes with nest-directed stops [6].

The neuronal mechanisms for the storage of an internal representation of the solar ephemeris during initial LW are still unknown. Computational models demonstrate that the CX circuitry is able to store spatial (heading) information [37] based on orientation cues from visual and other modalities [38]. This makes the CX a suitable candidate for a memory-based calibration of visual compass cues, which is also supported by the learning-dependent volume increase of the CX shown in our present study. The differences in the synaptic architecture and behavioural function of the CX and mushroom body visual pathways inferred from circuit analyses and ablation experiments suggest that view-based panoramic information is stored in the mushroom bodies, whereas directional information is processed in the CX [12,13,29,39,40]. It is not possible with the methods currently available to quantify distinct synapses or pre- and postsynaptic elements within subunits of the CX (as it is possible for synaptic MG in the mushroom bodies) for exploring the exact source of volume increase. This could potentially be due to an increase in the number of synaptic connections, but also to the extension of dendrites as shown in the mushroom bodies [29]. The CX is involved in several tasks related to spatial orientation like the directional control of movement [41,42], sun orientation [11], landmark orientation [43] and angular path integration [43]. The CX, therefore, is an important integration centre for multisensory orientation cues [44] making it a well-suited neuropil to combine polarization information with other directional cues, for example, terrestrial reference systems.

Due to the large numbers of Kenyon cells, the mushroom bodies in *Cataglyphis* brains are well-equipped to store many celestial snapshots. Many thousands of microcircuits in the mushroom body collar provide a suitable neuronal substrate for this [4,16]. Such snapshot-based learning mechanisms have been extensively studied in view-based learning of the visual panorama during hymenopteran LW and flights [45–47]. Furthermore, UV light is important for determining directions based on the visual panorama [48]. Therefore, UV light is crucial for different view-based navigational strategies, but it remains unclear whether polarized light information is also transferred to the mushroom bodies. Our results demonstrate that LW-related neuroplasticity in the mushroom bodies is dependent on polarization information suggesting that information about a changing polarization pattern is somehow registered in the mushroom bodies. This might be achieved via direct transfer by polarization-sensitive neurons or indirect feedback via modulatory information from other brain areas, which needs to be addressed in future physiological studies. Finally, for sky-compass calibration, the information about sky polarization dynamics needs to be combined with input from the endogenous clock, which also requires future investigation to fully understand the processes underlying the acquisition of an internal ephemeris [3].

In order to learn the rotation of the sky, animals need a geostable reference [2,5,36]. Experienced honeybees use the landmark panorama for re-calibrating the movement of celestial compass cues [49,50]. Since naive *C. nodus* use a magnetic compass during initial LW [8], our hypothesis is that the earth's magnetic field provides the geostable reference system with which the solar ephemeris is initially calibrated [3,8,51]. This would allow the ants to calibrate the celestial compass and to learn the landmark panorama simultaneously during initial LW. As we propose for *Cataglyphis*, migratory birds are able to calibrate their celestial compass based on magnetic compass information [52]. Birds use a wide range of different navigation cues for their long-distance migration and adapt the cue hierarchy according to their navigational needs [53]. Similarly, *Cataglyphis* ants switch between different navigation cues and value them differently throughout their foraging careers [1,54,55,56]. The brain of *C. nodus* contains multimodal integration centres [19,57] that allow for the convergence of orientation cues from different sensory modalities. To become a successful forager, compass information from the polarization pattern of the sky needs to be further integrated with input from the magnetic compass, sun position and internal clock [3]. The present results suggest that early experience of a moving polarization pattern together with a geostable magnetosensory input [8] during initial LW in the natural habitat provide the crucial multimodal sensory input necessary to trigger neuroplastic changes resulting in calibrations of the visual compass systems.

## Data accessibility

All processed data are supplied within the manuscript and electronic supplementary material [58]. Raw data (confocal image stacks of whole brains and 3D data) will be made available from the authors upon request.
